# Familial mediterranean fever and pauciimmune renal vasculitis: the role of IL-1 blockade (Anakinra)

**DOI:** 10.1186/1546-0096-9-S1-P5

**Published:** 2011-09-14

**Authors:** MS Camacho Lovillo, E Iglesias, O Neth, V Santa-María, A Sánchez, J Bernabeu

**Affiliations:** 1 Department of Pediatric Infectious Diseases and Immunodeficiencies; 2 Department of Pediatric Nephrology; 3 Department of Dermatology, Hospital Infantil Virgen del Rocío, Seville, Spain

## Background

Familial mediterranean fever (FMF) is an autoinflammatory disease been considered an autosomal recessive disease. 60% of patients are homozygotes. It can be associated with Henoch-Schönlein purpura and panarteritis nodosa. There is an increase in the diagnosis of heterozygosis form in FMF patients with other rheumatic diseases and it is believed that these genes could modulate the expression of digenic inheritance

## Case report

A four years old girl was admitted to our hospital in May 2007 with a 1 week history of fever, haematuria, abdominal pain, a polycyclic exanthema with white centre and Beau lines on her nails. She has been suffering for 2 years from periodic fever associated with tonsillitis and 3 months prior admission a febrile episode was accompanied with an urticarial like exanthema. Laboratory: WBC 36.580/microL (neutrophils 80%), Hb 9.7 g/dl, VSG 108 mm/h and PCR 150 mg/l; proteinuria 60 mg/m^2^/h, creatinine clearance 65,7 ml/min/1,73 m2. Serology (viral and bacterial), rheumatoid factor and ANA: negative; Ig G,A,M, and C4, C3 serum: normal. Skin biopsy : leucocitoclastic vasculitis ; Kidney biopsy : pauciimmune glomerulonephritis. Cytology of a bone marrow aspirate: normal. Genetic study: heterozygosity for V726A mutation (MEVF gen). Initial treatment with corticosteroids and cyclophospamide did not result in clinical remission and the later was replaced by colchicine with initial improvement. However symptoms persisted in episodes when corticosteroids were tapered. Therapy with Il-1R antagonist anakinra (2 mg/kg/day) was initiated in May 2008 and corticosteroids could be tapered and withdrawn successfully. She continues to suffer from recurrent but very mild hematuria and abdominal pain associated with low grade temperature whilst renal function remains normal.

## Conclusion

Pauciimmune vasculitis can be associated with FMF

Immunomodulation with Anakinra might be used in the management of patients with FMF who do not response on conventional treatment.

